# Efficacy of Antimicrobial Photodynamic Therapy for Treating Moderate to Deep Periodontal Pockets in Individuals with Type 2 Diabetes Mellitus: A Systematic Review and Meta-Analysis

**DOI:** 10.3390/dj13010021

**Published:** 2025-01-02

**Authors:** João Victor Soares Rodrigues, Mariella Boaretti Deroide, Wilton Mitsunari Takeshita, Valdir Gouveia Garcia, Rafael Scaf de Molon, Leticia Helena Theodoro

**Affiliations:** 1 Department of Diagnosis and Surgery, School of Dentistry, São Paulo State University (UNESP), Araçatuba 16015-050, SP, Brazil; joao.soares@unesp.br (J.V.S.R.); mariella.b.deroide@unesp.br (M.B.D.); wilton.takeshita@unesp.br (W.M.T.); rafael.molon@unesp.br (R.S.d.M.); 2 Latin American Institute of Dental Research and Teaching (ILAPEO), Curitiba 80710-150, PR, Brazil; vg.garcia@uol.com.br

**Keywords:** diabetes mellitus, periodontal disease, periodontal pocket, photochemotherapy, photosensitizing agents

## Abstract

**Background/Objectives:** Diabetes mellitus and periodontitis share a significant, bidirectional relationship. Diabetes raises the risk of periodontitis and influences its severity, impacting tissue repair and bone metabolism. Conversely, periodontal inflammation can disrupt glycemic control, further complicating this interlinked relationship. This systematic review aimed to evaluate the efficacy of antimicrobial photodynamic therapy (aPDT) as an adjunct to subgingival instrumentation (SI) in the treatment of periodontal pockets with a probing pocket depth (PPD) ≥ 5 mm in individuals with type 2 diabetes mellitus (DM2) and periodontitis. **Methods:** Using the PICOS framework, this review addressed the following question: “How does aPDT as an adjunct to SI compare to SI alone in treating periodontal pockets with PPD ≥ 5 mm in individuals with DM2 and periodontitis?” Databases searched included PubMed, Scopus, and Web of Science up to December 2024. Randomized clinical trials evaluating periodontal status and HbA1c levels in patients with DM2 undergoing periodontal therapy and experiencing SI were included. Patients who received adjunctive aPDT were compared to a control group that received SI alone. A meta-analysis was conducted illustrating treatment effects across groups. **Results:** After screening 117 studies based on titles and abstracts, three and four studies met the eligibility criteria for quantitative and qualitative analyses, respectively. The principal periodontal parameters assessed included PPD, clinical attachment level (CAL), plaque index (PI), and bleeding on probing (BOP). Forest plots for PD, BOP, PI, and CAL at baseline, three months, and six months revealed no statistically significant differences between the SI+aPDT group and the SI-only group. Glycated hemoglobin across treatment groups was not different. **Conclusions:** The combination of aPDT with SI provides limited clinical benefits in treating periodontal pockets with a PPD ≥ 5 mm in diabetic patients with periodontitis.

## 1. Introduction

Diabetes mellitus (DM) is a chronic endocrine disorder characterized by elevated blood glucose levels resulting from either insufficient insulin secretion, insulin resistance, or a combination of both factors [1]. In 2021, approximately 536.6 million individuals globally were living with diabetes, with Brazil ranking sixth worldwide in the number of cases among adults aged 20 to 79, totaling around 15.7 million people [2,3].

For individuals with type 2 diabetes mellitus (DM2), prolonged hyperglycemia promotes the formation of advanced glycation end products (AGEs), which play a role in triggering pro-inflammatory and oxidative stress responses. These AGEs interact with receptors (RAGE) on cells, affecting cellular function, reducing collagen synthesis, and impairing bone regeneration, ultimately interfering with tissue repair [4,5]. This metabolic disturbance is associated with various comorbidities, including cardiovascular, neurological, skeletal, renal, and periodontal diseases [6].

Periodontitis is a chronic, bacteria-driven inflammatory condition triggered by dysbiotic biofilm and an excessive immune response, resulting in damage to the structures that support teeth [7]. Clinical manifestations include gingival bleeding, attachment loss, alveolar bone deterioration, and the formation of periodontal pockets [8]. Periodontitis remains a global public health problem, with a combined prevalence of nearly 62% in dentate adults [8]. Several factors can modify its progression, such as rheumatoid arthritis [9], cardiovascular disease [10,11], pulmonary disease [12], DM [13,14], and inflammatory bowel disease. Notably, DM and periodontitis share a significant, bidirectional relationship: diabetes not only raises the risk of periodontitis but also influences its severity, impacting processes like tissue repair, bone metabolism, immune cell activity, and the composition and volume of gingival crevicular fluid, as well as the local blood supply [15]. Conversely, periodontal inflammation can disrupt glycemic control, further complicating this interlinked relationship [16,17].

Standard periodontal treatment focuses on removing bacterial buildup from tooth surfaces via non-surgical mechanical procedures, surgical methods, and maintenance strategies [18]. Non-surgical periodontal therapy, which includes subgingival instrumentation (SI), biofilm removal, and oral hygiene guidance, remains the gold standard for managing inflammation and infection, as well as reducing periodontal pockets with a probing pocket depth (PPD) ≥ 5 mm [18]. However, in patients with systemic conditions, conventional treatment may be less effective at fully eradicating pathogens, particularly in difficult-to-reach areas like furcation and deep periodontal pockets, which can serve as reservoirs for bacteria and lead to recolonization of treated sites [18]. To enhance non-surgical therapies and improve microorganism control in such areas, adjunctive treatments are often employed as systemic or topical antibiotics [19].

Antimicrobial photodynamic therapy (aPDT) is one such adjunctive approach, using a light-activated photosensitizing agent in the presence of oxygen to generate reactive oxygen species [20,21,22,23,24]. These cytotoxic species, including free radicals (via type I reactions) and singlet oxygen (via type II reactions), target various microorganisms. aPDT offers broad antimicrobial action against bacteria, fungi, and protozoa with minimal risk of resistance and no significant side effects, making it a valuable option for disinfecting root surfaces and treating residual periodontal pockets [25,26]. In this context, this review addressed the following question: “How does aPDT as an adjunct to SI compare to SI alone in treating periodontal pockets with PPD ≥ 5 mm in individuals with DM2 and periodontitis?”

## 2. Materials and Methods

### 2.1. Protocol Registration and PICO Strategy

This systematic review followed the Cochrane Collaboration guidelines [27] and adhered to the Preferred Reporting Items for Systematic Reviews and Meta-Analyses Protocols (PRISMA-P) for planning [28]. The review was registered in the PROSPERO under approval protocol CRD42023486429.

This review aimed to answer the following clinical question: how does aPDT as an adjunct to SI compare to SI alone in treating periodontal pockets with PPD ≥ 5 mm in individuals with DM2 and periodontitis? Following the PICO criteria, the study included the following.

Population: Adults of both sexes with DM2 and periodontitis;

Intervention: Use of aPDT as an adjunct to SI in periodontal pocket with PPD ≥ 5 mm;

Comparison: Non-surgical periodontal therapy;

Outcomes: Improvements in clinical periodontal measures, such as reduced PPD, bleeding on probing (BOP), and plaque index (PI).

### 2.2. Eligibility Criteria

Eligible studies included only randomized clinical trials (RCTs) involving adults over 20 years old with DM2 and periodontitis. These studies compared SI alone with SI combined with adjunctive aPDT, as well as aPDT in the treatment of periodontal pockets with PPD ≥ 5 mm. Only studies published in English were included, with requirements to report clinical outcomes, such as PPD, as the primary outcome, and clinical attachment level (CAL), BOP, and PI as secondary outcomes. Studies were excluded if they were in vitro or animal studies, lacked standardized group comparisons, lacked aPDT treatment, did not specify whether shallow, moderate, or deep pockets were treated, patients were undergoing initial periodontal treatment, or they consisted of case reports, case series, editorial letters, abstracts, reviews, or opinion articles.

### 2.3. Search Strategy

Two independent researchers (J.V.S.R. and M.B.D.) searched PubMed/MEDLINE, Scopus, and Web of Science for studies published over the entire period available in each database up to December 2024 by applying the eligibility criteria. Boolean operators (AND, OR) were used to combine keywords, thus refining the search strategy. All references were exported to Numbers software (Apple Inc., Cupertino, CA, USA), where duplicates were removed automatically using the software and manually. The search strategy incorporated a range of terms, such as (Diabetes Mellitus, Type 2) OR (Diabetes Mellitus, Noninsulin-Dependent) OR (Type 2 Diabetes) AND (Periodontitis) OR (Periodontal Pocket) AND (Photodynamic Therapy) OR (Photochemotherapy).

### 2.4. Selection Process

Two independent reviewers (J.V.S.R. and M.B.D.) screened titles and abstracts for eligibility. Full texts were obtained for references lacking sufficient title or abstract information for inclusion/exclusion decisions. Cohen’s kappa was employed to establish inter-rater reliability in the process of study selection, with an acceptable threshold value of 0.87. Any disagreements at any stage were resolved through discussion and mutual agreement with a third reviewer (L.H.T.). No differences in selection occurred between the two primary reviewers.

### 2.5. Risk of Bias Assessment

Bias risk was evaluated for each RCT individually using the “Cochrane Handbook for Systematic Reviews of Interventions” [29]. For each domain, studies were classified as having low risk, unclear risk (if insufficient information was available), or high risk of bias. Two authors (J.V.S.R. and M.B.D.) conducted these assessments independently, consulting a third author (L.H.T.) in cases of disagreement.

### 2.6. Data Analysis

A meta-analysis using a random-effects model was conducted to compare SI alone with SI combined with aPDT. The DerSimonian–Laird method was applied to account for heterogeneity across studies. Standardized mean differences (SMDs) were calculated based on means and standard deviations (SDs) for each outcome. Heterogeneity among the studies was assessed using Cochran’s Q test and quantified using the I^2^ statistic, with thresholds set at <25% for low, 25–75% for moderate, and >75% for high heterogeneity [30]. Statistical analyses were performed using Revman 5.3 software (Cochrane IMS, Copenhagen, Denmark).

## 3. Results

### 3.1. Study Selection

**Figure 1:**
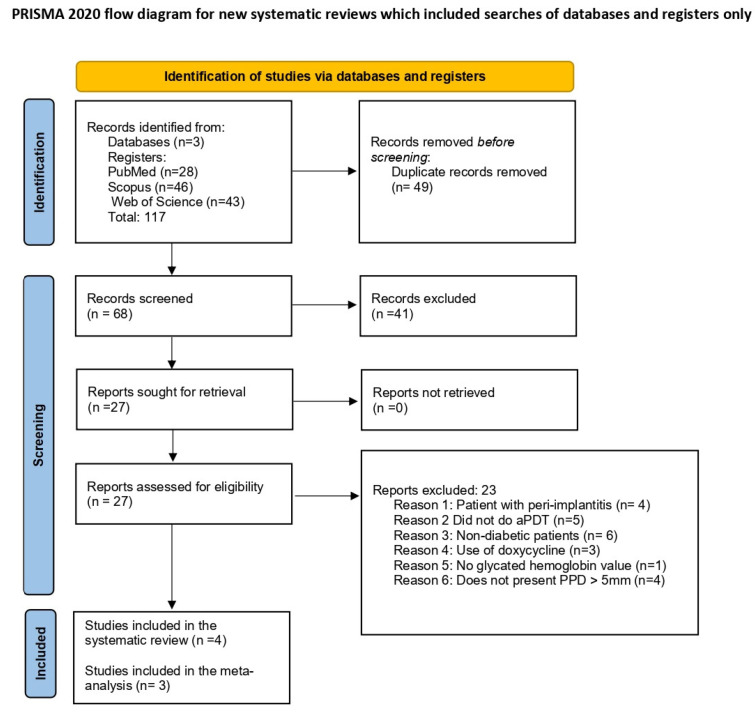
PRISMA flowchart of the included studies [31].

A total of 117 articles were initially identified through searches in the PubMed, Web of Science, and Scopus databases. After removing duplicates, 68 unique studies remained. Screening of titles led to the exclusion of 41 articles that did not meet the eligibility criteria. The abstracts of the remaining articles were then reviewed, resulting in 27 articles selected for full-text assessment. Following this in-depth review, 23 studies were excluded, leaving 4 studies eligible for data extraction and qualitative analysis and 3 studies for quantitative analysis (Figure 1).

### 3.2. General Characteristics of the Included Studies

This review incorporated four RCTs, three conducted in Brazil [20,21,23] and one in Slovenia [24]. Sample sizes varied, ranging from 12 to 25 participants per therapy group, with two studies utilizing a split-mouth design [20,23]. Participant ages ranged from a minimum of 21 to 41 years, and the oldest participants were between 68 and 75 years old. Each study focused on diabetic patients diagnosed with stage II to IV grade C periodontitis. One study compared curcumin (CUR) as an adjuvant to SI with aPDT as an adjunct to SI [20]. The other three studies examined aPDT as an adjunct to SI, using phenothiazines and a diode laser [21,23], while one study specifically investigated aPDT with indocyanine green and a diode laser [24].

### 3.3. Clinical Treatment Characteristics

In all four studies, SI were performed using the same technique, with aPDT included as an adjunct. Two studies employed an Indium Gallium Aluminum Phosphide (InGaAlP, 660 nm) diode laser with an optical fiber tip [21,23], while the third study utilized a Gallium Nitride Indium (InGaN) LED light source (465–485 nm) directed at the buccal and lingual surfaces [20], while the fourth study utilized a diode laser with 810 nm [24] (Table 1).

The study by Castro dos Santos et al. [23] evaluated 20 participants divided into two groups: Group 1, ultrasonic periodontal debridement (DPU), and Group 2 (DPU+aPDT). Both groups showed a statistical difference in PPD in the control group from 5.75 ± 0.91 mm at the beginning of the study to 3.47 ± 0.97 mm at 6 months and in the test group 6.15 ± 1.27 mm at 3.71 ± 1.63 mm. The CAL parameters at the beginning of the study ranged from 6.35 ± 1.27 to 4.61 ± 1.92 in 180 days. This difference was detected within the group, but it did not show a significant difference in the intergroup comparison.

In Ivanaga et al. [20], they assessed 25 participants divided into four groups: Group 1 (SI), Group 2 (SI+CUR irrigation), Group 3 (LED), and Group 4 (SI+aPDT). The aPDT group demonstrated marked improvements, with an increase in CAL to 4.95 ± 2.33 at 90 days and 5.46 ± 1.98 at 180 days. The PPD decreased significantly from baseline to 4.33 ± 1.78 mm by 180 days, with a notable reduction in deep pockets, highlighting the enhanced benefits of using aPDT as an adjunct to SI.

In the study by Cláudio et al. [21], 34 participants were split into two groups: SI alone and SI+aPDT. Both groups saw reductions in PPD and BOP and an increase in CAL, along with a decrease in the number of moderate pockets. Notably, deep periodontal pockets showed reductions in PPD by 5.14 ± 1.90 mm at 90 days and 4.46 ± 1.33 mm at 180 days, with a 20% decrease in high-risk progression in the SI group and a 31.25% reduction in the SI+aPDT group.

In Brinar et al. [24], they evaluated 24 participants divided into two groups: Group SI and Group SI+aPDT showed a statistical difference for the group SI+aPDT for BOP in addition to a smaller presence of pathogenic bacteria T. forsythia. In relation to CAL, it went from 3.7 ± 0.02 to 3.2 ± 0.2 in 3 months and PPD 3.3 ± 0.2 to 2.6 ± 0.2, presenting an intragroup statistical difference.

### 3.4. Glycemic Control and aPDT Efficacy

Four studies [20,21,23,24] did not observe significant reductions in glycated hemoglobin across treatment groups.

### 3.5. Bias Assessment

**Figure 2:**
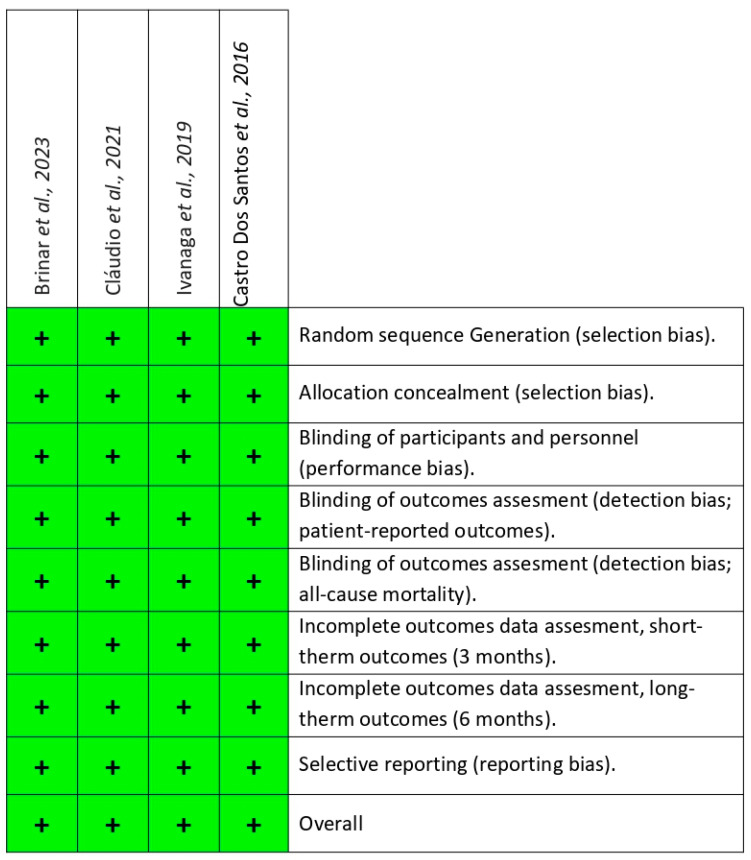
Cochrane risk of bias for each study (summary) [20,21,23,24].

Any discrepancies regarding study quality were thoroughly reviewed before presenting the bias summary, as illustrated. The assessment of the risk of bias of the selected studies is presented in Figure 2. In summary, the four chosen studies [20,21,23,24] were classified as low visual risk of bias because all of them provided sufficient information and details regarding each of the domains.

### 3.6. Data Analysis for Meta-Analysis

Of the 27 studies selected for full-text reading, three manuscripts were eligible for meta-analysis. Forest plots were used to graphically display effect sizes and 95% confidence intervals (CIs). A two-tailed *p* < 0.05 was used to determine statistical significance. Heterogeneity was assessed using Cochran’s Q test and quantified using the I^2^ index. The values of the analyzed variables presented *p* > 0.05 and heterogeneity (I2) less than 7%. All values analyzed comparing the SI group with the SI and aPDT groups presented *p* > 0.05.

### 3.7. Primary Meta-Analysis Outcomes

**Figure 3:**
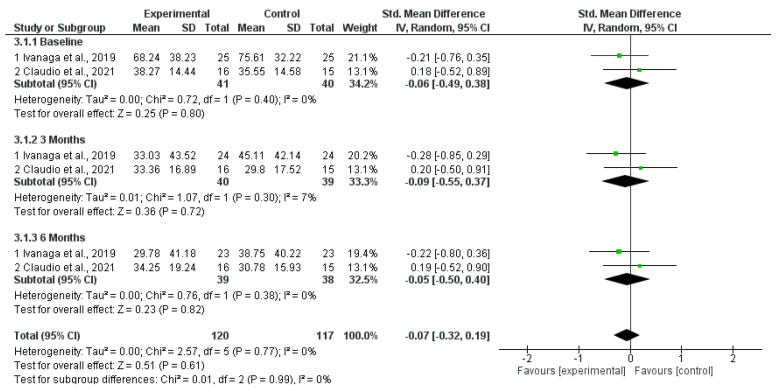
Forest plot for PI comparing adjuvant SI+aPDT versus SI at baseline of non-surgical periodontal treatment after 3 and 6 months of follow-up [20,21].

PI %: The meta-analysis showed no statistically significant differences in PI between the SI+aPDT group and the SI-only group at baseline, three months, or six months (Figure 3).

**Figure 4:**
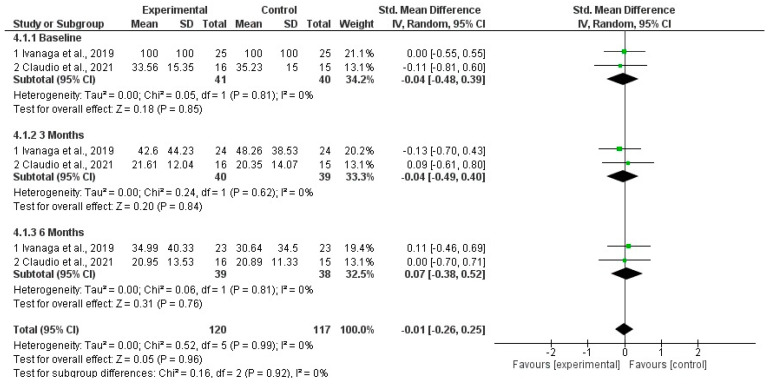
Forest plot for BOP comparing adjuvant SI+aPDT versus SI at baseline of non-surgical periodontal treatment with the beginning of treatment and after 3 months and 6 months of follow-up [20,21].

BOP %: Meta-analysis results for BOP at baseline, three months, and six months similarly showed no significant difference between the SI and SI+aPDT groups (Figure 4).

**Figure 5:**
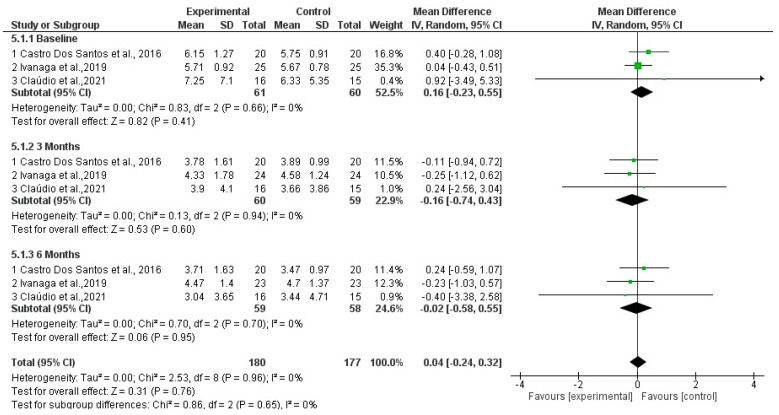
Forest plot for PPD comparing adjuvant SI+aPDT versus SI at baseline of non-surgical periodontal treatment after 3 months and 6 months of follow-up [20,21,23].

PPD %: Forest plots for PPD at baseline, three months, and six months revealed no statistically significant differences between the SI+aPDT group and the SI-only group (Figure 5).

**Figure 6:**
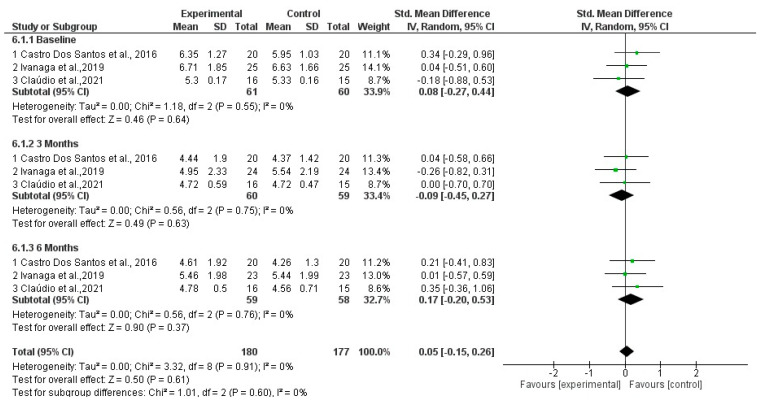
Forest plot for CAL comparing adjuvant SI+aPDT versus SI at baseline of non-surgical periodontal treatment after 3 months and 6 months of follow-up [20,21,23].

CAL %: Forest plots assessing CAL at baseline, three months, and six months indicated no significant differences between the SI+aPDT and SI groups (Figure 6).

## 4. Discussion

In this systematic review, we aimed to evaluate the hypothesis that aPDT, as an adjunct to SI, would yield superior clinical outcomes compared to SI alone in the treatment of periodontal pockets with PPD ≥ 5 mm in patients with DM2, thus fostering new perspectives for their clinical recommendations. Our data demonstrated that patients presenting with DM2 combined with periodontal pockets with PPD > 5 mm presented limited improvements in clinical periodontal parameters when aPDT was associated with SI. The levels of HbA1c were not significant decreased when aPDT was associated with SI in the included patients, suggesting that this adjunctive approach did not lead to improvements in glycemic control.

Individuals with DM face several metabolic changes that affect their immune response, making them more vulnerable to infections and inflammation. The presence of low-grade inflammation characterized by increased secretion of pro-inflammatory cytokines, chemokines, and prostaglandins in the serum may contribute to the severity of inflammatory chronic conditions, such as periodontitis [32]. Additionally, impaired connective tissue metabolism in diabetic patients—characterized by a decrease in both the function and number of fibroblasts—leads to lower collagen production and increased vulnerability to connective tissue destruction. This condition makes periodontitis, particularly in its severe forms, a contributing factor that can adversely affect glycemic control in these individuals [33].

DM2 contributes to periodontal disease progression by promoting inflammation through advanced glycation end products, which bind to RAGE receptors and enhance inflammatory signaling, impair cellular repair, and increase bone resorption [20]. Some studies have noted a decrease in gingival crevicular fluid AGEs following aPDT with SI, indicating potential benefits for diabetic patients [34]. Research suggests that periodontal therapy can reduce HbA1c levels by an average of 0.36%, an effect comparable to that of adding metformin as an adjunct therapy for metabolic control in patients with DM2 [35]. This connection implies that periodontal treatment not only enhances oral health but may also significantly support glycemic control [36]. However, few studies have examined the combined effects of aPDT with non-surgical periodontal treatment on HbA1c levels in diabetic patients [20,21,22,23,24,37,38]. Some evidence shows HbA1c improvement with this approach [38], while other studies indicate no additional benefit [20,21,22,23,24,37].

Non-surgical periodontal treatment, such as SI, has been shown to be effective in reducing PPD and BOP in patients with periodontitis [39]. However, the success of the treatment goes beyond the method used; it depends on an integrated approach that includes adequate debridement, effective maintenance therapy, and patient adherence to recommendations [40]. However, in cases where periodontal pockets persist after treatment, such as those with PPD ≥ 4 mm and BOP or PPD ≥6 mm, subsequent intervention becomes necessary. This may include surgical approaches or a combination with adjuvant therapies to improve outcomes [18]. Conversely, the clinical practice guideline for stage I–III periodontitis does not currently endorse the adjunctive use of aPDT, citing insufficient scientific evidence [18]. The European Federation of Periodontology based its recommendation on only two clinical studies, highlighting the limited evidence supporting aPDT as an adjunctive therapy due to the small number of controlled clinical trials available [18,41].

Some studies have investigated the impact of periodontal therapy on diabetic patients [20,21,22,23,24,42,43]. These studies explored various adjunctive approaches, including local antibiotic application [42], comparisons between surgical and non-surgical treatments [43], and the use of aPDT [20,21,22,23,24]. A recent systematic review on non-surgical periodontal treatment with antimicrobials in individuals with DM2 concluded that adding topical or systemic antibiotics provides only modest clinical benefits in managing periodontitis in diabetic patients [19], which parallel observations made in this systematic review.

aPDT relies on three main components: a photosensitizer, a light source (laser or LED), and oxygen. Together, they produce reactive oxygen species capable of eliminating various microorganisms, including bacteria, viruses, and fungi, without significant side effects and with a low risk of bacterial resistance [21]. Its basic principle is the combination of visible or near-infrared light, oxygen, and a photosensitizer capable of absorbing light and transferring energy or electrons to molecular oxygen. This process generates reactive oxygen species, which are toxic to periodontopathogenic microorganisms [44]. These reactive oxygen species disrupt the microbial cell membrane, impair protein function, damage nucleic acids, and break down bacterial DNA, which can affect both Gram-positive and Gram-negative bacteria [44].

Methylene blue and toluidine blue, both cationic phenothiazine dyes, are effective against Gram-positive and Gram-negative bacteria, as well as fungi [45]. Some studies have shown a significant reduction in the viability of periodontal pathogens, such as *Aggregatibacter actinomycetemcomitans*, *Porphyromonas gingivalis*, and *Tannerella forsythia,* after six months of treatment [46]. In terms of microbiological analysis, among the included studies, the study with one session of aPDT using indocyanine green showed a significant reduction in the levels of *Tannerella forsythia* when compared to SI alone [24]. However, another study with three sessions using methylene blue did not show a significant reduction in the levels of *Porphyromonas gingivalis* or *Prevotella intermedia* [21].

Several factors can influence the effects of aPDT, including the photosensitizer agent and its concentration, the light source, the irradiation parameters, and the pre-irradiation time [47]. One key factor that appears to impact aPDT’s efficacy is the choice of photosensitizer, along with its concentration and the duration of its contact with the tissue [48]. Among the primary photosensitizers used in aPDT, such as methylene blue, toluidine blue, curcumin, and indocyanine green, these agents are capable of penetrating infected tissues and releasing reactive oxygen species when exposed to light, generating singlet oxygen to eliminate pathogens [44].

Most studies have evaluated the effects of phenothiazines as photosensitizers combined with a 660 nm laser [21,23]. Its absorption is near an emission of 630 to 660 nm. In this review, methylene blue at a concentration of 10 mg/mL was used by Cláudio et al. [21], while Castro dos Santos et al. [23] used 0.005% methylene blue (µg/mL) as a photosensitizing agent, while Brinar et al. [24] evaluated the effect of indocyanine green at a concentration of 1 mg/mL combined with an 810 nm diode laser. The absorption peak of indocyanine green is near the emission maximum of 800 nm. Another study by Ivanaga et al. [20] used curcumin at a concentration of 100 mg/L. Curcumin is a naturally occurring lipophilic compound derived from Curcuma rhizomes known for its antibacterial, anti-inflammatory, and antioxidant properties with an absorption peak near the 420 nm emission.

It is important to note that the included clinical studies in this review used different types of photosensitizers (phenothiazines, curcumin, and green indocyanine) in combination with lasers or LEDs with varying wavelengths due to differences in absorption coefficients. In addition to the type and concentration of the photosensitizers, the irradiation parameters of each light source (LED or laser) also varied across the studies. The power of the light sources ranged from 60 mW to 250 mW, with energy densities ranging from 7.69 to 157 J/cm^2^. The number of sessions also varied among the studies. Among the studies included in this review, one conducted three sessions of aPDT with a 48-h interval [21], while the others performed a single application immediately after SI [20,23,24]. In the study that used three applications with methylene blue and a diode laser (660 nm), a reduction in BOP and PPD was observed in deep pockets 180 days post-treatment [21].

Several clinical studies have shown beneficial effects of using aPDT as an adjunct therapy for treating residual pockets in systemically healthy patients [49,50,51,52,53], while others have reported no added benefit [54,55]. Studies on systemically healthy patients examining the combination of aPDT with SI observed improvements in clinical parameters, such as gains in CAL [56] and reductions in PPD and BOP [57,58]. However, some studies found no significant advantage in adding aPDT to SI for enhancing clinical outcomes [44,59,60]. Recent research has also evaluated the efficacy of adjunctive aPDT in treating periodontal disease among systemically compromised patients, including those with DM2, with results indicating beneficial effects, particularly in reducing PPD [20,21,22,23] and BOP [20,21,22,24].

The included studies present a low risk of bias and applied the treatment to a similar sample (PPD > 5 mm), demonstrating high internal validity. On the other hand, two studies used a split-mouth model, limiting the number of patients included in the sample, which may reduce the external validity of the studies. Moreover, the differences among the photosensitizer used, the number of aPDT applications, and the regime of administration are factors that negatively impact the external validity of the studies.

Finally, it is important to mention that our study has certain limitations that should be acknowledged. First, the limited number of studies comparing periodontal clinical parameters across different analysis periods may affect the strength of our conclusions. Additionally, the results of the meta-analysis are constrained, particularly concerning the SI + aPDT combination, and should therefore be interpreted with caution. Nevertheless, the evidence presented in this review suggests that using aPDT as an adjunct to SI may offer modest improvements in periodontal parameters. This highlights the need for further research to confirm these effects and to assess their broader clinical relevance.

## 5. Conclusions

In conclusion, combining aPDT with SI in the treatment of periodontal pockets with a PPD ≥ 5 mm in diabetic patients provides modest clinical benefits. Although no significant benefits were observed when aPDT was combined with SI, further long-term, multicenter, randomized clinical trials are needed to confirm its efficacy and better understand its impact on systemic health.

## Figures and Tables

**Table 1:** General characteristics of the included studies.

Study Country	Type of Study	Number of Patients	Interventions	Study Duration	Results
Castro dos Santos et al. (2016) [23] Brazil	Split-mouth, randomized controlled clinical trial	20 individuals with DM2	After SI, periodontal pockets with PPD ≥ 5 mm were treated with a 0.005% methylene blue application. Following a 60 s waiting period, irradiation was performed using a diode laser (660 ± 10 nm) for 60 s at 60 mW, delivering an irradiance of 2.15 W/cm^2^, totaling 3.6 J and a fluency of 129 J/cm^2^. The optical fiber tip was positioned in the periodontal pocket area during the procedure.	6 months	Both groups showed a reduction in PPD after 180 days. However, all intergroup analyses revealed no significant differences in periodontal clinical parameters.
Ivanaga et al. (2019) [20] Brazil	Split-mouth randomized controlled clinical trial	25 individuals with DM2	After SI, periodontal pockets with PPD ≥ 5 mm were irrigated with 1 mL of curcumin solution. Following 1 min of irrigation, LED irradiation (465–485 nm) was applied for 60 s, with a power of 78 mW and a total energy of 7.69 J/cm^2^ on the buccal or lingual surfaces.	6 months	Reduction in PPD and BOP at three and six months; gain in CAL at three months compared to initial data.
Cláudio et al. (2021) [21] Brazil	Parallel randomized clinical trial	34 individuals with DM2	After SI, three applications of aPDT were performed on pockets with a PPD ≥ 5 mm. The pockets were irrigated with a 10 mg/mL methylene blue solution, and after 60 s, diode laser irradiation (660 ± 10 nm) was applied for 50 s at 100 mW, delivering a total energy of 4.7 J and an irradiance of 157 J/cm^2^, with the fiber optic tip placed in the periodontal pocket area.	6 months	Reduction in BOP at 90 and 180 days; decrease in the number of residual pockets at 90 and 180 days, reduced CAL in deep pockets (180 days).
Brinar et al. (2023) [24] Slovenia	Parallel randomized controlled trial	24 individuals with DM2	After SI, indocyanine green (1 mg/mL) was applied to pockets with a PPD ≥ 5 mm for 60 s, followed by diode laser irradiation (810 nm) at 250 mW.	3 months	A significant reduction in BOP was observed. However, comparison between groups did not show statistically significant differences in PPD, CAL, and gingival recession.

Abbreviations: DM2—diabetes mellitus type 2; SI—subgingival instrumentation; CUR—curcumin; aPDT—antimicrobial photodynamic therapy; PPD—probing pocket depth; BOP—bleeding on probing; CAL—clinical attachment level; GR—gingival recession.

## Data Availability

Data generated in this research project can be accessed by contacting the last author of this paper via email. They are stored electronically as Excel worksheets.
